# Cloning of K26 Hydrophilic Antigen from Iranian Strain of *Leishmania infantum*

**Published:** 2017-10

**Authors:** Bibi Razieh HOSSEINI FARASH, Mehdi MOHEBALI, Bahram KAZEMI, Homa HAJJARAN, Behnaz AKHOUNDI, Reza RAOOFIAN, Abdolmajid FATA, Majid MOJARRAD, Mohammad Kazem SHARIFI-YAZDI

**Affiliations:** 1. Dept. of Medical Parasitology and Mycology, School of Public Health, Tehran University of Medical Sciences, Tehran, Iran; 2. Center for Research of Endemic Parasites of Iran, Tehran University of Medical Sciences, Tehran, Iran; 3. Dept. of Biotechnology, School of Medicine, Shahid Beheshti University of Medical Sciences, Tehran, Iran; 4. Legal Medicine Research Center, Iranian Legal Medicine Organization, Tehran, Iran; 5. Dept. of Medical Parasitology and Mycology, School of Medicine, Mashhad University of Medical Sciences, Mashhad, Iran; 6. Zoonosis Research Center, Tehran University of Medical Sciences, Tehran, Iran

**Keywords:** Visceral leishmaniasis, *Leishmania infantum*, K26 immunodominant antigen

## Abstract

**Background:**

Visceral leishmaniasis (VL) caused by *Leishmania infantum* is the most severe form of leishmaniasis in Iran, which causes a high mortality rate in the case of inaccurate diagnosis and treatment. This study aimed to clone of K26 gene from Iranian strain of *L. infantum* and register the sequencing results in Genbank to facilitate the preparation a new K26 antigen for the detection of *L. infantum* infection.

**Methods:**

*L. infantum* was obtained from an infected domestic dog in Meshkin-Shahr area from northwestern Iran in 2015. Canine visceral leishmaniasis was confirmed by direct agglutination test (DAT), rK39 dipstick and parasitological methods. *L. infantum* was confirmed by N-acetyl glucosamine -1-phosphate transferase (nagt)–PCR and its sequencing. The band of interest for k26 form Iranian strain of *L. infantum* was purified by gel extraction kit after PCR amplification and then ligated into pBluescript II SK (+) and pET-32a (+), respectively. The sequences of recombinant plasmids were analyzed and submitted to Genbank.

**Results:**

The submission of rk26 nucleotide sequence was performed to the GeneBank/NCBI Data Base under accession number KY212883. The related gene was showed a homology about 99% to *L. chagasi* and *L. infantum* k26 gene, while the level of homology in comparison with different strains of *L. donovani* ranged from 84–94%.

**Conclusion:**

The successful rk26 cloning into an expression vector performed in this study could help to produce a new recombinant antigen for serodiagnosis of VL especially in areas where *L. infantum* is the main causative agent.

## Introduction

Leishmaniasis is one of the major health problems with more than 12 million new cases annually, worldwide (1). Different species of *Leishmania* as unicellular protozoan can lead various clinical forms of disease ranging from cutaneous leishmaniasis (CL) to diffused cutaneous, mucosal manifestations and occasionally, visceral leishmaniasis(VL). The latter one is the most severe form caused by *L. donovani* complex in the Indian subcontinent and East Africa, by *L. chagasi* in Latin America and by *L. infantum* in Iran and other Middle East countries, Europe, North Africa. Some authors believe *L. infantum* and *L. chagasi* are completely synonyms, until proposing the new classification for *Leishmania* genus (2,3). Northwestern and south of Iran are considered as endemic areas for VL where an increasing rate of newly infected cases have been reported, annually (4,5).

The fatality of symptomatic VL is estimated 100% without accurate diagnosis and treatment. The laboratory diagnosis of VL, a parasitic disease with high morbidity and mortality caused by *L. donovani* complex, is challenging. Domestic dogs and other wild canines have been known as the efficient reservoirs for human VL in all geographical zones of Iran, thus detection of canine VL is necessary for control and prevention of disease (5,6). Observation the parasite in aspirate materials or detection of anti-*Leishmania* antibodies using the serological techniques are the current methods for diagnosis of VL. The latter procedures use crude antigen preparations lacking in specificity. The recombinant antigen rK39 obtained from *L. chagasi* has proved a high sensitivity and specificity for the serodiagnosis by ELISA in the symptomatic form of human and canine VL, while sera of asymptomatic infection were generally negative with rK39 and have demonstrated a better sera reaction with *Leishmania* lysate (7). The commonly used methods of diagnosis have been based on detection of amastigotes in aspiration of the bone marrow, spleen, liver or the lymph nodes by Giemsa-stained slides and high-powered lens. These tests not only are known as invasive methods with non-desirable sensitivity but also require the skilled people who perform a biopsy and microscopic examination with special equipment (8).

Serodiagnosis of VL during an acute or subclinical form of the disease is typically exploited for anti-*Leishmania* antibody detection by using different methods. One of the most popular serology tests in countries, which suffer from VL, is direct agglutination test (DAT), but some limiting factors have been reported such as cross-reaction with some other infection in lower titers or variation in results by different species of antigen. In addition, after several years even after treatment, the titer of IgG antibody may detect in the serum samples (9). Too much effort to produce new different recombinant antigens has been applied for obtaining high sensitivity and specificity in the diagnosis of VL. However, rK39 protein from *L. chagasi* has shown reliable results in New World VL, its application for *L. infantum* and *L. donovani* in Old World VL including Iran has not demonstrated the sensitivity and specificity as high as reported for diagnosis of New world VL (10,11).

Recent studies on another new recombinant antigen K26 have shown a greater sensitivity in rK26 than rK39 in ELISA test, especially in the early stage of infection (12). Different results have been obtained during working with various species of *Leishmania* including *L. infantum* (13).

Although preparation of recombinant polypeptides related to Iranian *Leishmania* species have been attempted by Iranian researchers in last decade, there are no impressive follow-up or more studies to optimize appropriate antigens for the diagnosis of VL in human and dog (11,14). Prolonged process of cloning, sophisticated equipment is the major limiting factors affected on possibilities to investigate (15).

This study aimed to describe the cloning of K26 of Iranian *L. infantum* gene and register the sequencing result in Genbank to facilitate the preparation a new rK26 antigen to evaluate this protein on the diagnosis of VL in Iranian patients infected to VL in the next studies.

## Materials and Methods

### Ethical consideration

The Ethics Committee of Tehran University of Medical Sciences reviewed and approved this project. *L. infantum* was obtained from an infected domestic dog in Meshkin-Shahr area from northwestern Iran.

### Parasite and Culture

The used isolate was obtained from a symptomatic dog with hepatosplenomegaly, skin ulcers naturally, infected in Meshkin-Shahr, an endemic area, for VL in 2015. The infection was confirmed by direct agglutination test (DAT) and rK39 dipstick and then biopsy samples of liver and spleen were aseptically prepared for culture and direct parasitological method (16).

Promastigotes of *L. infantum* were cultured in RPMI-1640(Sigma-Aldrich) and 10% inactivated fetal bovine serum (FBS) in a sterile condition at 25 ± 1 °C. Promastigotes were kept at 20 °C after harvesting the organism in logarithmic phase and washing by phosphate-buffered saline (PBS), pH 7.2.

### N-acetylglucosamine -1-phosphate transferase (nagt) –PCR and Sequencing

According to the manufacturer’s instructions, DNA was extracted by the High Pure PCR template preparation kit (Roche Diagnostics GmbH, Mannheim, Germany). In order to determine the *Leishmania* species, a partial sequence of the nagt gene from the genomic DNA was amplified by using the primers (L1, forward: 5′-TCA TGA CTC TTG GCC TGG TAG-3′; and L4, reverse: 5′-CTC TAG CGC ACT TCA TCG TAG-3′). The PCR reaction carried out in a 25-μl volume, using 95 °C for 5 min, followed by 30 cycles of 94 °C for 1 min, 55 °C for 1 min and 72 °C for 90 sec by 96x thermocycler (Peq-lab, Germany). The band of interest for *Leishmania* spp. was observed by UV light in 1.5% agarose gel (Invitrogen, Life Technologies GmbH, and Germany) (16). The PCR product was sent to Pishgam Company to analyze partial sequence of nagt gene and then submitted to Genbank and received accession number.

### PCR amplification of L. infantum k26 gene

The primers designed based on sequence of K26 gene of *L. chagasi* (GenBank Accession number AF131228) were, forward, F, 5′- GGAGCCTACTGCACGAAGGAC-3′ containing a BamHI site and reverse, R, 5′- GTTGCCGGCAACCTGCTCC -3′ which contained a Xo1 site for k26 open reading frame of *L. infantum* (GenBank Accession number KT201383). PCR amplification for the desired fragment was performed by ABI DNA thermal cycler and a proof reading DNA polymerase (pfu) (Fermentas, Lithuania). The PCR reaction mixture contained 1.5 μl (5 pm) of each primer, 0.5 μl dNTPs and 0.5 μl MgSo4. The PCR program included 94 °C for 5 min, followed by 30 cycles at 94 °C for 1 min, 68 °C for 1 min, 72 °C for 2 min, and a final extension for 20 min.

### Cloning of PCR products and DNA sequencing

Purification of PCR product with expected length was performed by DNA extraction kit and ligated into pBluescript II SK (+) digested with EcoRV. The ligation product was transformed into *E. coli*, DH5alpha competent cells. The recombinant plasmids were extracted from white clones containing inserts and then digested by BamH1 and Xho1 restriction enzymes. The digested and purified product was ligated into the pET-32a (+) after double digestion with BamH1 and Xho1 enzymes in frame with histidine-binding protein. Finally, the new recombinant plasmids were extracted from six clones and submitted for sequencing with T7 terminator forward and reverse primers after PCR screening and plasmid extraction. The sequence of nucleotides was analyzed in comparison with data in the BLAST search method.

## Results

Amplification of partial sequence of nagt gene produced band approximately 1450–1460 bp in PCR for the sample obtained from the infected dog. The identity of the isolate showed 99%–100% homology with *L. infantum* spices registered in GenBank. The sequence data were deposited in GenBank database under accession number KT201383.

**Fig. 1:**
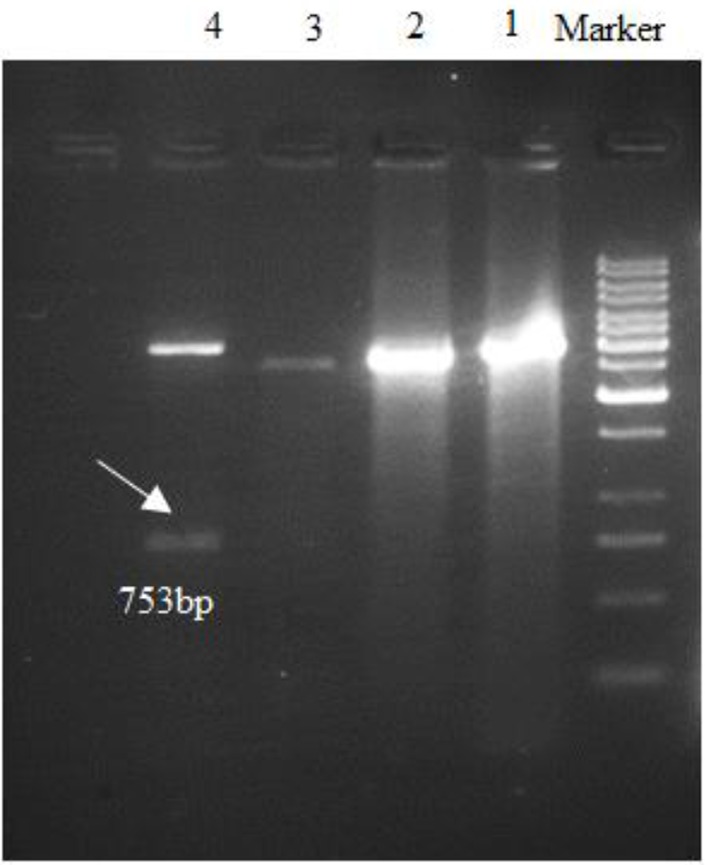
Double digestion of plasmid results with BamHI and XhoI after cloning into pBluescript II SK (+). Right to left: M, molecular-weight standard 1kbp; lanes 1, 2, 3, non-cloned plasmids for k26, respectively; lane 4, k26 cloned plasmid.

**Fig. 2:**
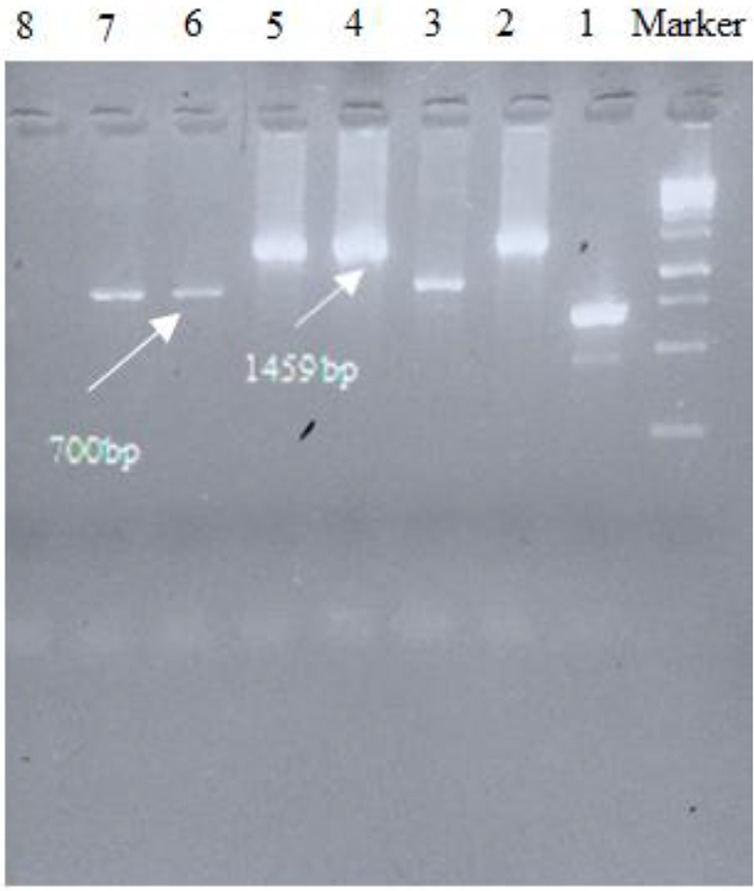
PCR results of screening of cloned plasmids after ligation into pET-32a (+) with T7 terminator primers. Right to left: M, molecular-weight standard 1kbp; lanes 2, 4, 5, cloned plasmids for k26, respectively; lanes 3, 6,7, non-cloned plasmids; lane 8, Negative control.

The coding region for *L. infantum* (accession number KT201383) k26 gene was amplified and showed a band in 753 bp which cut from the gel and extracted DNA cloned in pBluescript II SK (+), then subcloned in pET-32a (+) expression vector and the sequence was subsequently analyzed (Fig. 1,2). The submission of rk26 nucleotide sequence was performed to the Gen-Bank/NCBI Data Base under accession number KY212883. The related gene was compared with other rk26 sequences in the database using BLAST program and showed a homology about 99% to *L. chagasi* and *L. infantum* k26 gene, while the level of homology in comparison with different strains of *L. donovani* ranged from 84%–94%.

## Discussion

Observation of parasite in biopsy samples has remained gold standard method for diagnosis of VL, whereas the several disadvantages have been reported. Use of various antigens in serologic tests for accurate diagnosis of VL has been applied in several studies during recent decades (8). However, the accessibility and acceptable results of serologic methods even in situations with lack of facilities have made them as the first option for the screening of VL, the sensitivity and specificity depend on the methodology and assay. Furthermore, sensitivity is related to the type of methods, while the specificity has a close relation to the antigens employed (6). Specificity of antigens in this series of tests plays an important role because of cross-reactivity of antibody in other infective diseases such as cutaneous leishmaniasis, malaria, tuberculosis and toxoplasmosis, which can be present where VL is endemic (13).

The rK39, rK28, rK9 and rK26 have been proposed among new recombinant antigens (17). K26 is one of the recommendations with high specificity and sensitivity along with rK9 or rK39 either alone, although, the performance completely depends on source and purity of the antigen (13). In our study, an Iranian species of *L. infantum* has been employed obtained from an infected dog from Meshkin-Shahr where is endemic for VL in Iran.

The vector used at first stage in this research was pBluescript II containing antibiotic resistance sequence to ampicillin and a sequence within a LacZ controlled gene in multiple cloning sites designed for blue and white screening expressed in bacteria. Most previous studies worked on rk26 cloning or other recombinant *Leishmania* antigens have been cloned the desired sequence in this vector (1,7,13).

The pET vectors are known as a powerful system for the cloning and expression of recombinant proteins in *E. coli* (18,19). The pET 32a (+) was used at the second stage of cloning in this study for high-level expression of peptide sequences and containing cleavable His•Tag® and S•Tag™ sequences in cloning site for detection and purification (20). The pET as an expression vector was used to produce rK9 and rK26 protein and PDI2 recombinant protein, respectively while PQE 80L commercial vector was applied for rK26 protein expression (1,13,21).

High percentage of GC (more than 56%), more stacking interactions and secondary structures in this gene made some problems in amplification solved by increasing the melting temperature and adjusting the magnesium concentration in set up process (22). A high denaturing temperature was used to optimize PCR amplification for GC rich sequence (23).

A further downside is that K26 gene insert contains repeated sequences which caused improper annealing during PCR amplification of the band of interest or in ligation process (24,25). The main recommendation for this status is codon optimization that could help by reducing repetition throughout the DNA sequence, while the codons will preserve the desired amino acid. Moreover, it could result in a high quantity in protein expression process in *E. coli* (26).

In order to this purpose, it will be tried for gene synthesis, after cloning the k26 gene in pET 32a (+) and gene sequencing, but several specialized companies stated that not be able to gene synthesize due to repeated nucleotide sequences. Repetitive DNA was problematic in cloning, maintaining in bacteria, amplifying, sequencing and gene synthesis (23,25).

In the first evaluation of rK26 by ELISA was performed in Iran, have been reported a high sensitivity about 96.8% and specificity 100% among VL-confirmed patients (11). Whereas, this finding is in disagreement with other sensitivity results demonstrated a low sensitivity for this antigen (1,9,27,28). This controversy has been considered due to differences in methods used and K26 antigen applied where *L. chagasi* is not the main cause of VL.

Our sequencing finding on *L. infantum* k26 gene has shown the similarity of K26 antigen is high with *L. infantum* and *L. chagasi* (99% identity), while there is more discrepancy with *L. donovani*. In addition, the recombinant k26 gene of the used isolate was compared to *L. infantum* LON49 applied and it was determined only a single nucleotide difference in sequence (13). It is seriously recommended preparing *Leishmania* antigen from origin species for diagnosis of VL.

## Conclusion

The successful rk26 cloning performed in this study could help to start producing a new recombinant antigen for serodiagnosis of VL especially in where *L. infantum* is the main causative agent for this disease. Furthermore, this would make this possible to compare and discuss the real sensitivity of rK26 prepared by Iranian *L. infantum* and its application along Iranian rk39 protein to diagnose VL in Iran.

## Ethical considerations

Ethical issues (Including plagiarism, informed consent, misconduct, data fabrication and/or falsification, double publication and/or submission, redundancy, etc.) have been completely observed by the authors.

## References

[1] Bhatia A Daifalla NS Jen S Badaro R Reed SG Skeiky YA (1999). Cloning, characterization and serological evaluation of K9 and K26: two related hydrophilic antigens of *Leishmania chagasi*. Mol Biochem Parasitol, 20;102(2):249–61. 10498181 10.1016/s0166-6851(99)00098-5

[2] Dantas-Torres F (2006). *Leishmania infantum* versus *Leishmania chagasi*: do not forget the law of priority. Mem Inst Oswaldo Cruz, 101(1):117–8. 16699722 10.1590/s0074-02762006000100024

[3] Pattabhi S Whittle J Mohamath R (2010). Design, development and evaluation of rK28-Based Point-of-Care tests for improving rapid diagnosis of visceral leishmaniasis. PLoS Negl Trop Dis, 14;4(9): e822. 20856856 10.1371/journal.pntd.0000822 PMC2939046

[4] Mohebali M Hajjaran H Hamzavi Y (2005). Epidemiological aspects of canine visceral leishmaniosis in the Islamic Republic of Iran. Vet Parasitol, 15;129(3–4):243–51. 15845279 10.1016/j.vetpar.2005.01.010

[5] Mohebali M (2013). Visceral leishmaniasis in Iran: Review of the epidemiological and clinical features. Iran J Parasitol, 8(3):348–58. 24454426 PMC3887234

[6] Srivastava P Dayama A Mehrotra S Sundar S (2011). Diagnosis of visceral leishmaniasis. Trans R Soc Trop Med Hyg, 105(1):1–6. 21074233 10.1016/j.trstmh.2010.09.006 PMC2999003

[7] Badaró R Benson D Eulálio MC (1996). rK39: A cloned antigen of *Leishmania chagasi* that predicts active visceral leishmaniasis. J Infect Dis, 173(3):758–61. 8627048 10.1093/infdis/173.3.758

[8] Banu SS Ahmed B-N Shamsuzzaman AKM Lee R (2016). Evaluation of recombinant K39 antigen and various promastigote antigens in sero-diagnosis of visceral leishmaniasis in Bangladesh. Parasite Epidemiol Control, 1(3):219–28. 10.1016/j.parepi.2016.07.003 PMC5991841 29988192

[9] Sundar S Rai M (2002). Laboratory Diagnosis of Visceral Leishmaniasis. Clin Diagn Lab Immunol, 9(5):951–8. 12204943 10.1128/CDLI.9.5.951-958.2002 PMC120052

[10] Sivakumar R Sharma P Chang K-P Singh S (2006). Cloning, expression, and purification of a novel recombinant antigen from *Leishmania donovani*. Protein Expr Purif, 46(1):156–65. 16172002 10.1016/j.pep.2005.07.027

[11] Farahmand M Nahrevanian H (2016). Application of recombinant proteins for serodiagnosis of visceral leishmaniasis in humans and dogs. Iran Biomed J, 20(3):128–34. 26883952 10.7508/ibj.2016.03.001 PMC4949976

[12] Rosati S Ortoffi M Profiti ME (2003). Prokaryotic expression and antigenic characterization of three recombinant *Leishmania* antigens for serological diagnosis of canine leishmaniasis. Clin Diagn Lab Immunol, 10(6):1153–6. 14607883 10.1128/CDLI.10.6.1153-1156.2003 PMC262443

[13] Farajnia S Darbani B Babaei H Alimohammadian MH Mahboudi F Gavgani AM (2008). Development and evaluation of Leishmania infantum rK26 ELISA for serodiagnosis of visceral leishmaniasis in Iran. Parasitology, 135(9):1035–41. 18561868 10.1017/S003118200800454X

[14] Taran M Mohebali M Modaresi MH Mamishi S Mojarad M Mahmoudi M (2007). Preparation of a K39sub recombinant antigen for the detection of *Leishmania infantum* antibodies in human: a Comparative study with an immunochromatographic test and direct agglutination. Iran J Parasitol, 2(2):25–33.

[15] Akhoundi B Mohebali M Babakhan L (2010). Rapid detection of human *Leishmania infantum* infection: A comparative field study using the fast agglutination screening test and the direct agglutination test. Travel Med Infect Dis, 8(5):305–10. 20971441 10.1016/j.tmaid.2010.09.001

[16] Hajjaran H Mohebali M Teimouri A (2014). Identification and phylogenetic relationship of Iranian strains of various *Leishmania* species isolated from cutaneous and visceral cases of leishmaniasis based on N-acetylglucosamine-1-phosphate transferase gene. Infect Genet Evol, 26:203–12. 24911282 10.1016/j.meegid.2014.05.026

[17] Elmahallawy EK Martinez AS Rodriguez-Granger J Hoyos-Mallecot Y Agil A Mari JMN (2014). Diagnosis of leishmaniasis. J Infect Dev Ctries, 13;8(8):961–72. 25116660 10.3855/jidc.4310

[18] Taheri T Seyed N Mizbani A Rafati S (2016). *Leishmania*-based expression systems. Appl Microbiol Biotechnol, 100(17):7377–85. 27435294 10.1007/s00253-016-7712-4

[19] Rosano GL Ceccarelli EA (2014). Recombinant protein expression in *Escherichia coli*: advances and challenges. Front Microbiol, 5:172. 24860555 10.3389/fmicb.2014.00172 PMC4029002

[20] Merck Millipore (2016). pET-32a (+) DNA - Novagen | 69015. Available from: http://www.emdmillipore.com/US/en/product/pET-32a+-DNA-Novagen,EMD_BIO-69015

[21] Ali D Abbady A-Q Kweider M Soukkarieh C (2016). Cloning, expression, purification and characterization of *Leishmania tropica* PDI-2 protein. Open Life Sci, 11(1):166–176.

[22] Reina O (2015). Problems amplifying GC-rich regions? Problem Solved! Trinity College of Dublin, the Ireland Available from: http://bitesizebio.com/24002/problems-amplifying-gc-rich-regions-problem-solved/

[23] Sahdev S Saini S Tiwari P Saxena S Singh Saini K (2007). Amplification of GC-rich genes by following a combination strategy of primer design, enhancers and modified PCR cycle conditions. Mol Cell Probes, 21(4):303–7. 17490855 10.1016/j.mcp.2007.03.004

[24] Godiska R Mead D Dhodda V (2010). Linear plasmid vector for cloning of repetitive or unstable sequences in *Escherichia coli*. Nucleic Acids Res, 38(6): e88. 20040575 10.1093/nar/gkp1181 PMC2847241

[25] Hommelsheim CM Frantzeskakis L Huang M Ülker B (2014). PCR amplification of repetitive DNA: a limitation to genome editing technologies and many other applications. Sci Rep, 23;4:5052. 24852006 10.1038/srep05052 PMC4031481

[26] GeneScript (2016). Codon Optimization - Increase Protein Expression. Available from: http://www.genscript.com/codon-opt.html

[27] Rosário EY do Genaro O França-Silva JC (2005). Evaluation of enzyme-linked immunosorbent assay using crude *Leishmania* and recombinant antigens as a diagnostic marker for canine visceral leishmaniasis. Mem Inst Oswaldo Cruz, 100(2):197–203. 16021309 10.1590/s0074-02762005000200015

[28] da Costa RT França JC Mayrink W Nascimento E Genaro O Campos-Neto A (2003). Standardization of a rapid immunochromatographic test with the recombinant antigens K39 and K26 for the diagnosis of canine visceral leishmaniasis. Trans R Soc Trop Med Hyg, 97(6):678–82. 16117962 10.1016/s0035-9203(03)80102-5

